# Availability of Audiology Services in Assisted Living Communities in Florida

**DOI:** 10.1093/geroni/igaa057.272

**Published:** 2020-12-16

**Authors:** Daniel Pupo, Hillary Rouse, Lindsay Peterson, Kathryn Hyer

**Affiliations:** University of South Florida, Tampa, Florida, United States

## Abstract

Florida has one of the largest populations of older adults in the U.S., and as a result the state also has a high prevalence of hearing loss. Given the growth of assisted living as a housing option for older adults, the purpose of this study was to determine the availability of audiology services in assisted living communities (ALCs) across Florida. Data on ALC location, characteristics and audiology service availability were collected from the Florida Agency for Health Care Administration (AHCA). County socioeconomic data were collected from the U.S. Department of Labor. Logistic regression and chi2 tests were used to examine the relationship between county socioeconomics and whether an ALC provided audiology services. We found that of the 3090 ALCs in Florida, audiology services were present in only 57 (3.2%). ALCs with audiology services were significantly more likely to be located in counties with a higher education level and a higher average income. This suggests a shortage of ALCs with audiology services in counties where residents have fewer resources. The results are concerning, given that individuals with fewer resources are less able to pay for audiology services on their own and evidence showing that poor hearing health late in life impacts individuals’ health and quality of life. Policy implications will be discussed, including the need for more ALCs to provide audiology services in counties with fewer resources. One possible solution is tele-audiology, which would enable a single audiologist to diagnose and prescribe hearing aids to patients in underserved areas.

